# From 1957 to Nowadays: A Brief History of Epigenetics

**DOI:** 10.3390/ijms21207571

**Published:** 2020-10-14

**Authors:** Paul Peixoto, Pierre-François Cartron, Aurélien A. Serandour, Eric Hervouet

**Affiliations:** 1 Univ. Bourgogne Franche-Comté, INSERM, EFS BFC, UMR1098, Interactions Hôte-Greffon-Tumeur/Ingénierie Cellulaire et Génique, F-25000 Besançon, France; paul.peixoto@univ-fcomte.fr; 2 EPIGENEXP Platform, Univ. Bourgogne Franche-Comté, F-25000 Besançon, France; 3 CRCINA, INSERM, Université de Nantes, 44000 Nantes, France; Pierre-Francois.Cartron@univ-nantes.fr (P.-F.C.); aurelien.serandour@ec-nantes.fr (A.A.S.); 4 Equipe Apoptose et Progression Tumorale, LaBCT, Institut de Cancérologie de l’Ouest, 44805 Saint Herblain, France; 5 Cancéropole Grand-Ouest, Réseau Niches et Epigénétique des Tumeurs (NET), 44000 Nantes, France; 6 EpiSAVMEN Network (Région Pays de la Loire), 44000 Nantes, France; 7 LabEX IGO, Université de Nantes, 44000 Nantes, France; 8 Ecole Centrale Nantes, 44300 Nantes, France; 9 DImaCell Platform, Univ. Bourgogne Franche-Comté, F-25000 Besançon, France

**Keywords:** epigenetics, history, DNA methylation, histones, epitranscriptomics

## Abstract

Due to the spectacular number of studies focusing on epigenetics in the last few decades, and particularly for the last few years, the availability of a chronology of epigenetics appears essential. Indeed, our review places epigenetic events and the identification of the main epigenetic writers, readers and erasers on a historic scale. This review helps to understand the increasing knowledge in molecular and cellular biology, the development of new biochemical techniques and advances in epigenetics and, more importantly, the roles played by epigenetics in many physiological and pathological situations.

## 1. From Genetics to Epigenetics

Until the XVIII century, the preformation theory stated that aptitudes and biological differences are determined by a god and that individual characters start at conception and are frozen. This theory was fought by Darwin’s theories and Kant, suggesting that the environment was strictly involved in phenotype modifications. This led the definition of the concept of evolution. Mendel’s principles in 1865, the isolation of the DNA molecule in 1869 and, about one century later, the resolution of the double helix structure of DNA in 1959, established the global principles of genetics and heredity. The developmental biologist Conrad H. Waddington (1905–1975) coined the word epigenetics to summarize a new branch of biology which focuses on the links between gene and protein expression [1]. In 1930, it was reported in *Drosophila* that the location of the gene *white* in heterochromatin or euchromatin was responsible for its activation or repression. These observations suggested that the local nucleus environment regulated gene expression [2]. In 1957, Waddington proposed the famous epigenetic landscape, in which a ball, symbolizing a cell, could follow different paths due to the roughness of the surface (which means intra- and extracellular environmental influences) [3]. During the mid-1970s to 1980s, the identification of the high mobility group (HMG) proteins permitted us to understand that specific proteins other than histones, known since 1884, may have an architectural role in chromatin and could influence phenotype expression.

**Figure 1:**
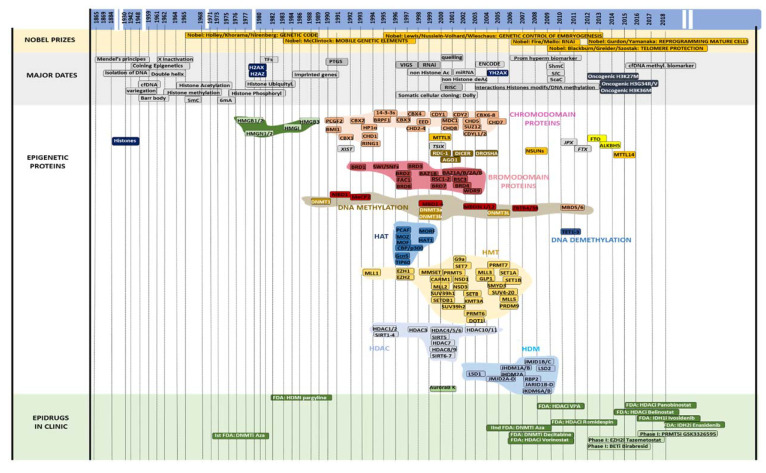
Chronology of epigenetics. From top to bottom: Dates (blue): from 1865 to today; Nobel Prizes (salmon) associated with major genetic or epigenetic discoveries; major dates (gray): important discoveries concerning DNA and chromatin; epigenetic proteins (white): year of identification or main writers, readers and erasers of epigenetics (a color code has been associated to proteins involved in a same pathway, e.g., blue for HAT (histone acetyl transferase)); epidrugs in the clinic (green): use of main epidrugs in clinical trials.

In the 1980s, several groups identified the requirement of a fusion of both a male gamete genome and a female gamete to generate viable fecundation and embryogenesis. This brought to light the existence of “imprinted genes” which are regulated specifically with regard to maternal or parental inheritance [4,5,6]. This process explained the high difficulties in obtaining clones after the transfer of a nucleus from an adult somatic cell into an enucleated egg, a concept that was considered impossible until the publication of Wilmut et al. in 1997 and the creation of the sheep Dolly [7]. This was followed by thousands of cloned animals in numerous different species and the generation of bimaternal and bipaternal mice in 2018, although these animals presented severe defects [8]. Indeed, even if the general organization of DNA was approximately understood quite early in mid-XX century, the boom of epigenetics arrived much later during the 1990s and 2000s with the full flow of cloning and biochemical techniques which allowed for the identification of specific enzymes, writers and erasers of epigenetic marks. The most studied and known epigenetic markers, DNA methylation (5mC) and post-translational histone modifications, were rapidly identified following the resolution of the DNA double helix structure. Indeed, DNA methylation was first described in 1965, while histone methylation, acetylation, phosphorylation, ubiquitylation, sumoylation and ADP ribosylation were reported from 1962 to 1977 [9,10]. The role of these modifications has been difficult to understand. Indeed, the late identification of enzymes catalyzing or erasing these markers permitted the performance of genetic and biochemical experiments, particularly in yeast models that progressively permitted the clarification of the biological significance of these modifications. All these epigenetic events and the identification of the main proteins involved in epigenetics are summarized in a chronology picture (Figure 1).

## 2. DNA Methylation

5mC is the most stable epigenetic marker whose negative role in gene regulation and in heterochromatin maintenance has been studied previously. The discovery of writers and erasers of DNA methylation took a very long time: from 1988 (cloning of DNA methyl transferase 1 (DNMT1), the first described writer of 5mC) to at least 2010 (identification of the TET family, erasers of 5mC) [11,12,13,14,15,16,17]. DNMT1, the first identified eukaryotic methyltransferase, has been purified and cloned in the lab of Timothy Bestor (University of Columbia). This team then collaborated with the lab of Rudolf Jaenisch (Whitehead Institute) to knock-out the *DNMT1* gene, revealing that this gene was essential for transposon silencing, X inactivation and imprinted gene regulation and, therefore, that DNA methylation writing is involved in many different biological processes. DNMT1 catalyzes the maintenance of DNA methylation following DNA replication to support the accurate transmission of this epigenetic mark to future daughter cells during mitosis. Identification of DNMT1 just preceded the discovery of the main family members of 5mC binding proteins (MeCP2, MBD1–4 from 1989 to 1998) which specifically recognize 5mC and coordinate gene repression [18,19,20,21,22]. Indeed, the lab of Adrian Peter Bird (University of Edinburgh) was the first to identify the recruitment of MeCP2 to methylated CpG and its involvement in gene repression. A.P. Bird showed that the blockade of DNA methylation identification by MeCP2 was associated with Rett syndrome. These discoveries revealed that DNA methylation was read by specific proteins, leading to a repressive signal, and that the disruption of these mechanisms was associated with human disorders [23].

Ten years after DNMT1, de novo DNMT3A and DNMT3B (1998) were identified and explained the acquisition of DNA methylation on both strands of DNA independently of DNA replication, a phenomenon particularly important for gene expression regulation during embryonic development and aberrant gene repression in many diseases, including cancers [12]. Indeed, the Bestor team identified the very rare immunodeficiency, centromeric region instability, facial anomalies (ICF) syndrome which is mainly caused by mutations in the *DNMT3B* gene, showing that DNA methylation writing during development is essential [24].

A few years later, DNMT3L was described and shown to participate in the activation of *de novo* DNMTs and DNMT-including complexes, but DNMT3L itself lacks catalytic activity [13,25]. Although DNA demethylases were known for decades in prokaryotes, such proteins were actively searched for years in mammals. Indeed, in 2009, a convincing DNA demethylation process was identified in mammals with the rediscovery of 5hmC, 40 years after a forgotten observation made in *Trypanosoma* [26]. Then, the observation of 5fC and 5caC showed that DNA demethylation could be an active process, which led to the identification of specific DNA demethylases when the TET (Tet-eleven 1, 2 and 3) family was described in 2010 in the lab of Yi Zhang (Harvard Medical School) which was a revolution in the field of epigenetics [17].

Moreover, in 2003, cooperation between DNA methylation and histone modifications for gene repression was proposed by the characterization of different complexes: (i) MBD1, CAF1 and HP1, (ii) MBD1, SUV39H1 and HP1 and (iii) SUV39H1, HP1b and DNMTs [27,28,29]. DNA sequences recognized by DNMT are not specific. For example, DNMT3A and DNMT3B are present on A/T/C, T/A/C, A/T, T/G/A, C, G, C/G and A/G DNA sequences, suggesting that protein interactions with DNMTs are involved in specific DNA methylation [30,31]. The first evidence in favor of this hypothesis was reported in 2002 in the lab of Pier Guiseppe Pelicci (European Institute of Oncology), showing that the oncogenic PML-RAR (promyelocytic leukemia-retinoic acid receptor) protein regulated target promoters via specific interactions and recruitment of DNMT1 and DNMT3A [32]. A few years later, in 2005 and 2006, specific interactions of DNMTs with the transcriptional factors P53, c-MYC or PU.1 confirmed that genes were additionally regulated by the specific recruitment of DNMTs [33,34,35]. In 2009 and 2010, dozens of putative transcriptional factor (TF)/DNMT interactions were reported, suggesting that the specific DNA methylation is probably mainly due to this process [36,37].

## 3. Protein Acetylation

From 1992 to 2005, many different proteins presenting a bromodomain, a term historically from the protein coded by the *brm* gene in *Drosophila melanogaster* [38], were reported. Indeed, bromodomain-containing proteins, whose first member was BRD1 [39], contain a domain of about 100 residues which specifically recognizes acetylated lysines in histone tails and favors gene transcription by reorganizing local chromatin.

Despite the early description of histone acetylation in 1968 [40,41,42], and its role in local chromatin opening, by neutralizing positive charges of lysines and consequently decreasing histone–DNA interactions, writers of acetylation called histone acetyltransferases (HATs) remained unknown for a long time [43]. Indeed, it took almost 30 years, and the emergence of both molecular cloning techniques and the biochemical purification of proteins to identify and characterize eight different HATs containing an acetylase domain, in less than 2 years (1996–1998) [44,45,46,47,48,49]. The C. David Allis lab (Rockefeller University) was the first team to identify and purify an HAT that laid the cornerstones for the understanding how histone post-translational modifications are performed and regulate gene expression. Moreover, this team was a pioneer in the understanding of crosstalk between the same histone tails or different histones, leading to the notion of a “histone code” governing local gene expression.

Some proteins with histone deacetylase activity (histone deacetylase, HDAC) were first identified in 1995 and are associated with proteins containing a deacetylase domain. In 7 years (1995–2002), 18 different HDACs were reported [50,51,52,53,54,55,56,57,58,59,60,61,62,63,64,65] and divided into four classes based on their sequence homology of yeast, catalytic domain organization and their cellular localization. Class I (HDAC1, 2, 3 and 8) with a simple structure containing a deacetylase domain with short N-Ter and C-ter extensions, IIa (HDAC4, 5, 7 and 9), IIb (HDAC6 and 10) and IV (HDAC11) are considered as classical HDACs and have a zinc-dependent active site; class III (SIRTUINs: SIRT1–7) have an NAD-dependent active site. Moreover, some HDAC proteins appeared mostly localized in nuclei, others transit between the nucleus and cytoplasm and others are predominantly present in cytoplasm, showing that roles played by these enzymes are more varied than initially believed. Indeed, since 1997, a lot of non-histone targets have been reported, and some researchers have renamed these enzymes lysine deacetylases (KDACs), which were first described for the acetylation-induced conformational change of P53 which significantly improved its DNA-binding capacities [66]. Similarly, deacetylation of non-histone proteins was then described. For example, deacetylation of P53 by an HDAC1-containing complex was reported in 2000 confirming that acetylation and deacetylation of proteins are not strictly associated with chromatin regulation [67]. Indeed, a new nomenclature of chromatin-modifying enzymes has been proposed to clarify the chromatin-dependent and -independent roles of these proteins [68].

## 4. Protein Methylation

Similarly, the roles of histone methylations, markers identified in 1962, were studied a long time before the identification of writers and erasers of these markers. Histone methylations occur on different lysines and arginines of histones and may concern mono-, di-, and trimethylation on the same residue. Moreover, dimethylation of arginines could be symmetrical (me2s) or assymetrical (me2a).

According to target residue, the level of methylation and symmetry, the methylated marker could be interpreted as a permissive or repressive marker of gene transcription (for example, local H3K4me3 and H4R3me2a are favorable to transcription, while H3K27me3 and H4R3me2s inhibit transcription).

Chromodomain (chromatin organization modifiers)-containing proteins (described from 1991 to 2005) [69,70,71,72,73,74,75,76,77,78,79,80,81,82,83,84,85,86,87,88,89,90,91,92] present a domain of about 40–50 amino acids that specifically recognize chromatin, and particularly histone methylations and regulate gene expression positively or negatively. Although the role of chromodomain-containing proteins in gene repression was suspected since the early 1990s, the link between histone methylation and chromodomain was not identified before 2001, with the role of the heterochromatin protein 1 (HP1) protein [93]. Indeed, the lab of Joel Eissenberg (Saint Louis University) (2000) first proposed that HP1 may serve as a cross-linker, linking nucleosomal DNA and non-histone protein complexes to form higher-order chromatin structures [94], showing that some proteins may detect specific histone methylation and thus lead to gene repression. HP1 identified and cloned in mammals in 1993 is a member of a homologous protein family initially described in *Drosophila* and previously associated with heterochromatin binding, nuclear localization and gene silencing [95]. HP1 was also the first chromodomain-containing protein to explain gene repression, which is observed after translocation of an active gene in the heterochromatin environment, a phenomenon called position effect variegation in *Drosophila* [96], known from the 1950s but unexplained for decades [97]. HP1 contains two different kinds of chromodomain: a classical N-terminal chromodomain and one C-ter domain called the chromo shadow domain [69]. Both of these domains, as for other chromodomain-containing proteins, recognize and interact with other proteins to regulate heterochromatin structure. As early as 2000, it was reported that the chromo shadow domain interacts with a consensus pentapeptide present in specific protein partners [98], and in 2001, that these chromodomains recognize H3K9 methylation [99]. Indeed, chromo domain-containing proteins are divided into three classes. Proteins, such as HP1 which present an N-terminal chromo domain followed by a chromo shadow domain, belong to the first class. The second class includes proteins with a single chromo domain, whereas the third class includes proteins with paired tandem chromo domains. Identification of these proteins permitted us to better understand how epigenetic markers really regulate gene expression according to the histone code.

Contrary to HATs and KDACs which are not specific to a single residue, methylation of histones is specifically catalyzed on a particular residue and the redundancy of activity is quite limited. For these reasons, the identification of histone methyl transferases (HMTs) responsible of each methylation took 12 years (1993–2005) [100,101,102,103,104,105,106,107,108,109,110,111,112,113,114,115,116,117,118,119,120,121,122,123,124,125,126]. Although some proteins have been known to be involved as early as the 1990s in gene repression, their histone methylase activity was identified much later. Indeed, both enhancer of zeste homolog 1 and 2 (EZH1 and EZH2) were cloned in 1996 [101,104] and described as members of the gene repressor Polycomb, homolog of *Drosophila*, complex. However, the H3K27 HMT activity of these both proteins was not characterized before 2002 (EZH2) and 2008 (EZH1) [127,128,129]. Moreover, a higher level of complexity was found in 2004 when it was reported that two Polycomb members belonging to the PRC2 complex, embryonic ectoderm development (EED) and SUZ12, which interact with EZH2, are required for H3K27me3 activity [130].

HMTs contain a SET domain comprising about 130 amino acids, whose name codes for the HMT activity of the first three *Drosophila* HMTs were originally reported as: (Su(var)3–9 **S**uppressor of variegation 3–9), **e**nhancer of zeste (EZ) and **t**rithorax (Trx). The SET domain possesses catalytic activity towards the ε-amino group of lysine residues for mono-, di- or trimethylation using the methyl donor S-adenosyl methionine (SAM) as a cofactor. Similarly to acetylation and deacetylation activity, non-histone methylation targets of HMT have been reported for numerous proteins since 2004 (see review [131]), showing that, as seen for writers and erasers of acetylation a few years earlier, these enzymes are not specific to histones and are, for example, involved in the regulation of gene expression by controlling the activity of transcriptional factors such as P53, which is stabilized by a SET9-mediated methylation [131,132].

Surprisingly, the discovery of erasers of histone methylation occurred much later. Although, an enzymatic demethylation of N-methylated calf thymus histones by an unknown mechanism was reported in 1973 [133], histone methylation was considered for a long time as a permanent marker. Indeed, it was proposed that methylation was removed by cutting the histone tail [134] or by exchanging the methylated histone with a variant histone [135,136,137,138]. The existence of an active histone demethylase (HDM) was established in 2004 by the lab of Yang Shi (Harvard Medical School) with the resolution of the molecular activity of lysine-specific histone demethylase (LSD1), initially called KIAA0601 [139]. This discovery broke the dogma and many different HDMs were then rapidly identified. Indeed, the LSD1 family comprises two members (LSD1 and LSD2) which correspond to homologs of amine oxidases (containing a C-ter oxidase domain (AOD) that demethylates histones by a flavin adenine dinucleotide (FAD)-catalyzed oxidation of the methylated H3K4 and thus produces formaldehyde responsible of demethylation. From 2004 to 2009, more than 15 different HDMs were identified [140,141,142,143,144,145,146,147,148]. Another family of HDM was discovered in 2006 [147] containing a Jumonji-like domain C terminus (JmjC) and includes proteins with 2-oxoglutarate- and iron Fe(II)-dependent dioxygenases that use these cofactors in the presence of oxygen to hydroxylate the methylated lysine and notably produce unstable carbinolamine that spontaneously reacts to generate formaldehyde that demethylates the lysine.

## 5. Other Post-Translational Histone Modifications

As described before, other post-translational histone modifications such as histone ubiquitination, sumoylation or ADP ribosylation have been reported. Histone ubiquitination has been implicated in transcriptional regulation and DNA damage response (for review [149]). Indeed, mono-ubiquitination of H2A inhibits transcription by repressing H3K4me2 methylation.

Histone sumoylation, which consists of the addition of a small ubiquitin-related modifier (SUMO) protein on a histone via a process very closely related to the enzyme cascade involved in protein ubiquitination, may also modulate gene expression. Histone H4 binds to the E2 conjugating enzyme and is sumoylated in an E1 (SUMO-activating enzyme) and E2-dependent manner that mediates gene silencing through the recruitment of a histone deacetylase and HP1 [150].

Histones can also be ADP-ribosylated in Asp/Glu residues. This post-translational modification catalyzed by ADP-ribose transferases (ARTs) is required for DNA damage repair. However, the precise contribution of these modifications in DNA damage response and repair remains unclear [151].

## 6. Histone Variants

Predominant variants of histone H2A, H2AX and H2AZ, have been reported since 1980 [152]. H2AX represents about 10% of total H2A but its role in DNA repair was found only in 2002 with the identification of the phosphorylated form of H2AX, γH2AX (which could rise to 10% of total H2AX), and the kinases responsible of this phosphorylation [153]. The work of William M. Bonner (NIH) and his team brought major contributions in this field. Indeed, γH2AX rapidly associates with double-strand breaks and signals for DNA repair. Although the H2AZ variant is less abundant than H2AX, and not associated with a specific process, it recently appeared that H2AZ is affected by many different classical post-translational modifications, such as classical histones, and this variant, notably via its acetylated form, is particularly associated with transcriptional starting sites and enhancers of genes involved in stem cell biology (see review [154]).

Besides natural variants of histones, for the last few years, several mutated forms have been reported and associated with cancers, and lead to the concept of oncohistones. Different forms of histones are present in cells; for example, H3.1 is incorporated during the S-phase and is present throughout the genome, whereas H3.3 is cell cycle-independent and more specifically associated with the promoters that it regulates. Indeed, H3.1K27M, H3.3K27M, H3.3K36M and H3G34R/V were reported in 2012 and 2013 [155,156]. Indeed, both H3K27M and H3K36M block the HMT responsible for the specific methylation of this lysine, resulting in a global loss of the methylated mark in these cancers [157]. On the contrary, the effects of H3G34R/V are restricted to chromatin sites presenting nucleosomes, containing the mutant.

## 7. Epitranscriptomics: The Area of Non-Coding RNA

Since the beginning of the 1990s, a phenomenon of gene co-suppression was reported in plants following the introduction of a transgene [158,159]. A well-known experiment reported the appearance of white petunia variants in a purple population. This process was called transgene-induced post-transcriptional gene silencing (PTGS) in plants and quelling in fungi [160] but the molecular mechanism remained unknown for years. In plants, PTGS can also be provoked by viruses expressing host genes in a process called virus-induced gene silencing (VIGS) [161]. The RNA interference process, called RNAi, was first identified in 1998 in the nematode *Caenorhabditis elegans* by the team of Craig Cameron Mello (previously of the University of Massachusetts Medical School). C.C. Mello and his collaborator Andrew Fire were awarded a Nobel Prize for this revolutionary discovery. [162]. RNAi leads to the processing of a dsRNA into a 21–22 dsRNA, a small interfering RNA (siRNA) that both targets and destroys the complementary mRNA, leading to gene silencing. Proteins involved in this process have been identified since 1999 [163,164,165,166,167]. Indeed, both RDE-1 (RNAi-defective 1) and Dicer are indispensable for RNAi. Dicer family members are responsible for dsRNA processing and contain a helicase domain, a PAZ (Piwi/Argonaute/Zwille) domain, two RNAse III domains and one or two dsRNA-binding domains [167]. The multiprotein complex guided by siRNA and targeting mRNA destruction was described in 2000 and called the RNA-induced silencing complex (RISC) [168,169]. In 2001, a specific category of non-coding RNA was identified in vertebrates, miRNA, showing that the inhibition of gene expression was a physiological mechanism in mammals. The characterization of miRNA highlighted that gene expression was controlled at an unexpected level [170].

The field of long non-coding RNA (lncRNA) began in the 1990s with the identification of the lncRNA *XIST* (1991) [171] and its major role in X inactivation, a phenomenon described in 1961 by Marie Lyon [172] and the previous identification of the Barr body in 1948, but whose molecular mechanisms remained unknown until the discovery of *XIST*. The complexity of the lncRNA world increased rapidly, with the identification of several other lncRNAs also involved in X inactivation during later decades (*Tsix*, *Ftx*, *Jpx*) and thousands of others involved in different gene regulation systems [173,174,175]. More and more evidence suggests that many lncRNAs are involved in gene regulation by interfering with other non-coding RNAs but also via specific roles in the targeting of multi-protein complexes on chromatin.

## 8. Epitranscriptomics and RNA Methylation

Until now, more than 160 RNA modifications have been reported. Among them, RNA methylation appeared to be frequent and the most studied. However, although the methylation of RNA had been reported since the early 1970s by several groups, it took decades before the identification of its roles in cells due to the difficulties for many years to manipulate RNA *in cellulo* [176]. Indeed, m6A represents more than 80% of methylated residues in RNA and is present in about one per 2000 residues, more specifically in conserved consensus sites G/A, G/A, A, C, A/C/U. Methyltransferase-like 3 (METTL3) was the first RNA methyl transferase discovered in 1997, a long time before the second member METTL14 in 2014 [177,178,179]. METTL3 contains a SAM-binding site and a catalytic site called the CMII domain. Similar to the late identification of HDM, two different RNA demethylases were reported quite recently (in 2012 and 2014) [180,181]. Indeed, ALKBH5 contains an iron-binding motif and an α-ketoglutarate interaction domain involved in the RNA demethylation activity. In 2012, a role of m6A as a docking site was established for the recruitment of RNA-binding proteins (ELAV1, YTHD2, YTHD3) which could further recruit additional protein partners [182]. Similarly, m5C of RNA, a common epitranscriptomic marker identified for the first time in 1974 [183], is present in both mRNA and non-coding RNA where it regulates translation. However, the roles of this methylation are largely unknown and the identification of writers of this marker began very recently. Indeed, contrary to DNMT2, whose role in the methylation of C38 tRNA^Asp^ was reported in 1998 [184], the first member of the NSUN (NOP2/Sun RNA Methyltransferaase) m5C RNA methylase family was identified in 2000s [185]. In 2019, specific methylation of miRNA was reported in gastrointestinal cancers, suggesting that this process could be involved in specific gene expression in diseases [186].

## 9. Epidrugs: Towards Indispensable Therapies

The anticancer properties of azacitidine were first reported in 1964 [187] and the first clinical trial began in 1967. This drug was FDA approved in 1971 but its role as a DNMT inhibitor (DNMTi) was ignored for 13 additional years (see review [188]). Indeed, the pharmacologic effects of high-dose azanucleosides are supposed to be mainly responsible of the cytotoxicity of azanucleotide–DNMT adducts formed in DNA that could mask the effect of DNA demethylation via the clonal expansion of resistant cells. Due to high toxicities and the relative ignorance of roles played by epigenetics in pathologies for decades, azacitidine was rejected by the FDA and it took more than 3 decades to be FDA approved again, in 2004, for the treatment of patients with myelodysplastic syndrome (MDS). This molecule became the first drug approved against this pathology [189]. The lab of Moshe Szyf (McGill) was the first to relate DNA methylation modifications and links to pathologies, in particular, in cancers. Moshe Szyf predicted early on that epidrugs would be essential for modulating gene expression in cancer and he founded the first pharma lab to develop potential clinical epidrugs. In 2006, decitabine was also FDA approved, another DNMTi supposed to present fewer side effects at low doses.

Similarly, biological HDAC inhibitor (HDACi) effects were observed well before the identification of their role as HDAC inhibitors. Indeed, in 1971, DMSO was reported as a differentiation agent and, a few years later, bisamide, a precursor of suberonyl anilide hydroxyamic acid (SAHA), with a similar structure to DMSO, presented similar effects. Trichostatin A (TSA) was the first molecule whose role was identified as an HDACi in 1990 [190]. Concomitantly to decitabine, vorinostat was FDA approved in 2006 for the treatment of T cell lymphoma [191]. Rapidly, several HDACis were also approved for different diseases. Indeed, in the last 5 years, tens of clinical trials tested many other epidrugs in numerous pathologies, alone and mostly in combination, including HMTi and BETi (Bromo and extra-Terminal inhibitors). There is no doubt that epigenetics is now in the clinical arena and could be largely involved in patient care in the near future.

## Abbreviations

ARTs	ADP-ribose transferases
BRD1	Bromodomain-containing 1
DNMT	DNA methyl transferase
EZH2	Enhancer of zeste homolog
HAT	Histone acetyltransferase
HDAC	Histone deacetylase
HDM	Histone demethylase
HMG	High-mobility group
HMT	Histone methyl transferase
HP1	Heterochromatin-1
JmjC	Jumonji-like domain C terminus
KDAC	Lysine demethylase
lncRNA	Long non-coding RNA
LSD1	Lysine-specific histone demethylase
MBD	Methyl-binding domain
METTL	Methyltransferase like
RISC	RNA-induced silencing complex
RNAi	RNA interference
SAM	S-adenosyl methionine
SUV39H1	Suppressor ff variegation 3-9 homolog 1
TF	Transcriptional factor
